# Thermal Energy Storage and Mechanical Performance of Crude Glycerol Polyurethane Composite Foams Containing Phase Change Materials and Expandable Graphite

**DOI:** 10.3390/ma11101896

**Published:** 2018-10-04

**Authors:** Nuno Vasco Gama, Cláudia Amaral, Tiago Silva, Romeu Vicente, João Araújo Pereira Coutinho, Ana Barros-Timmons, Artur Ferreira

**Affiliations:** 1 CICECO—Aveiro Institute of Materials, 3810-193 Aveiro, Portugal; nuno.gama@ua.pt (N.V.G.); claudiaamaral@ua.pt (C.A.); tiagomsilva@ua.pt (T.S.); jcoutinho@ua.pt (J.A.P.C.); 2 Department of Chemistry of University of Aveiro, 3810-193 Aveiro, Portugal; 3 RISCO, 3810-193 Aveiro, Portugal; romvic@ua.pt; 4 Civil Engineering of University of Aveiro, 3810-193 Aveiro, Portugal; 5 Escola Superior de Tecnologia e Gestão de Águeda, 3750-127 Águeda, Portugal; artur.ferreira@ua.pt

**Keywords:** polyurethane foams, crude glycerol, phase change materials, expandable graphite, thermal energy storage, numerical simulations

## Abstract

The aim of this study was to enhance the thermal comfort properties of crude glycerol (CG) derived polyurethane foams (PUFs) using phase change materials (PCMs) (2.5–10.0% (*wt/wt*)) to contribute to the reduction of the use of non-renewable resources and increase energy savings. The main challenge when adding PCM to PUFs is to combine the low conductivity of PUFs whilst taking advantage of the heat released/absorbed by PCMs to achieve efficient thermal regulation. The solution considered to overcome this limitation was to use expandable graphite (EG) (0.50–1.50% (*wt/wt*)). The results obtained show that the use of PCMs increased the heterogeneity of the foams cellular structure and that the incorporation of PCMs and EG increased the stiffness of the ensuing composite PUFs acting as filler-reinforcing materials. However, these fillers also caused a substantial increase of the thermal conductivity and density of the ensuing foams which limited their thermal energy storage. Therefore, numerical simulations were carried using a single layer panel and the thermal and physical properties measured to evaluate the behavior of a composite PUF panel with different compositions, and guide future formulations to attain more effective results in respect to temperature buffering and temperature peak delay.

## 1. Introduction

One of the primary roles of external building envelopes is to assure good indoor thermal comfort conditions, by keeping the inside of buildings cool in the summer and warm in the winter. Hence, the capacity of the materials used to store/release thermal energy is essential [1,2,3,4]. Rigid PU foams (PUFs) are an important class of materials due to their outstanding thermal insulation properties which, compared with other insulation materials, are highly competitive. Moreover, if heat storage materials are additionally incorporated into PUFs, the heat loss to and the gain from surroundings, will be reduced. Therefore, the energy saving will be much more efficient [5,6,7,8].

In the last years, this concept has found growing interest as a result of the rise of a new class of materials: the so-called phase change materials (PCMs). PCMs, also called latent heat-storage materials, can store and release a significant amount of thermal energy within a small temperature range. According to their phase change states, they fall into three groups: solid–solid PCMs, solid–liquid PCMs and liquid–gas PCMs. In solid–liquid PCMs, the energy is absorbed by the breakdown of the bonds responsible for the solid structure. When the materials cool down, the latent heat previously absorbed is released to the surroundings and the PCMs return to its solid state [6,9,10,11,12,13]. Taking advantage of the heat storage ability of PCMs, Aydın and Okutan [14] incorporated fatty acid ester-based in rigid PUFs and reported that the total heat absorption capacity of ensuing foams was improved up to 34% when 22.6% (*wt/wt*) of PCM was used.

Nonetheless, in general, the low thermal conductivity of commonly used paraffin based PCMs requires the use of large and expensive heat transfer surfaces to improve it, which is considered a major drawback [15]. Additionally, in view of the price of PCMs, it would not be viable to consider loading PUFs with high amounts PCMs at large scale. Furthermore, their low thermal conductivity, combined with that of PUFs tends to further limit their application as thermal regulation materials. In that sense, the thermal conductivity of PUFs must be increased, and one option to achieve this is filling the polymer with conductive agents. Thermal conductive carbon based materials such as expandable graphite (EG) present several advantages over the usual metal fillers due to lower costs, corrosion resistance, less material requirement for percolation, and ease of processing [16]. Moreover, the addition of EG, besides enhancing the mechanical properties, allows improving the reaction to fire of the ensuing composites, which in the case of PUFs containing paraffin derived PCMs can be an important issue [15,17,18].

Besides improving the thermal conductivity of composites via the incorporation of thermal conductive materials, the amount of PCM must be minimized due to price constraints and the impact it would have on flammability and mechanical properties. Therefore, numerical simulations have been developed to optimize the amount of PCMs to achieve thermal comfort with the best environmental contribution [19].

Whilst the use of PUFs filled with PCM and EG to enhance the thermal efficiency of building per se is a contribution towards sustainability, the use of raw materials from renewable resources in the production of PUFs can be considered a further contribution. Indeed, this approach has been widely investigated and polyols derived from acid liquefied or oxypropylated biomass have been used to replace petroleum based polyols in the synthesis of PUFs [20,21,22,23,24,25,26,27]. More recently, crude glycerol (CG) has also been used in the production of PUFs [17,28,29,30,31,32]. Actually, the successful replacement of petrochemicals by CG in the production of PUFs has the potential to reduce costs and environmental impact.

In the present work, the enhancement of thermal energy storage and mechanical properties of CG derived PUFs were assessed. The approach followed was to add EG to the PUF and PCM composite to increase the thermal conductivity of the system to take advantage of PCMs’ ability to store/release energy. The resulting foams were fully characterized and numerical simulations were carried out to study the composites performance as energy comfort materials.

## 2. Materials and Methods

### 2.1. Chemicals

**Scheme 1:**
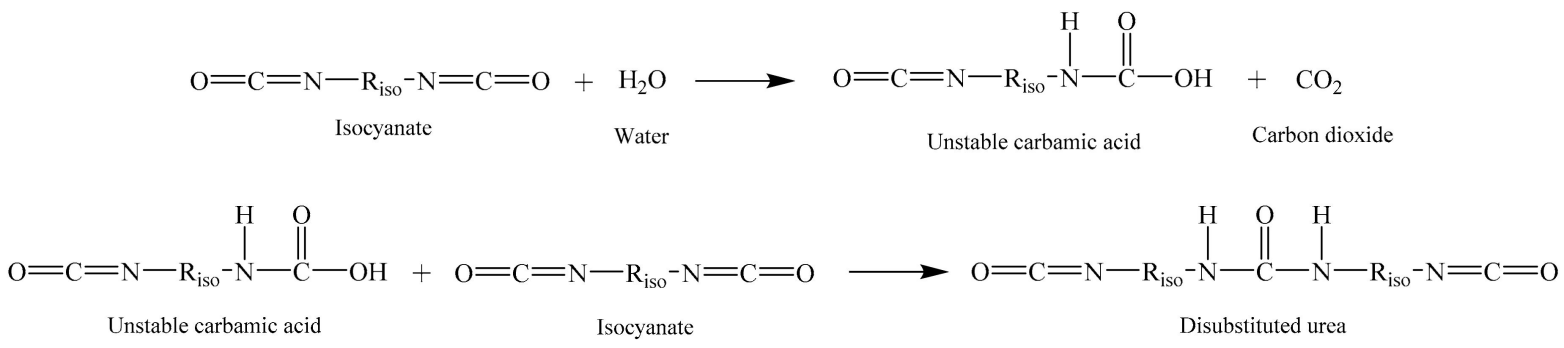
Reaction scheme of the isocyanate with water [33].

The foams studied were produced using EG and a polymeric isocyanate in the presence of a catalyst, a surfactant and a blowing agent. CG was kindly supplied by Bioportdiesel and presented a water content of 1.6% ± 0.01, an *AV* of 23.1 ± 0.2 mg_KOH_·g^−1^ and an *OH_number_* of 399.0 ± 4.7 mg_KOH_·g^−1^. The polymeric isocyanate Voranate M229 MDI with a NCO content of 31.1% (weight percent free isocyanate content), with a functionality of 2.7, a viscosity of 190 mPa·s (at 25 °C) and an isocyanate equivalent of 135 was kindly supplied by Dow Chemicals. Tegostab B8404, a polyether-modified polysiloxane with a density of 1.045–1.065 g·cm^−3^ (at 25 °C), was used as silicone surfactant and was supplied by Evonik. Polycat 34, a tertiary amine with a density of 0.84 g·cm^−3^ (at 25 °C), was used as catalyst and supplied by Air Products. Distilled water was used as blowing agent, which upon reaction with the isocyanate groups yields carbon dioxide, the blowing gas which is actually responsible for the foam expansion, and disubstituted ureas, as illustrated in Scheme 1 [33]. 

A microencapsulated paraffin wax (Micronal DS 5008 X) with a density of 250–350 kg·m^−3^ and a melting point of 26.2 °C was supplied by BASF and used as PCM. EG (EG GHL PX 95) with a thermal conductivity of 0.290 W·m^−1^·K^−1^ (at room temperature) and a density of 543.6 kg·m^−3^ was supplied by LUH. 

### 2.2. Characterization of Crude Glycerol

The determination of the water content was carried out using a KF 756 Coulometer for Karl Fisher titration, based on ISO 14897:2002 standard. The sample was analyzed using Hydranal (Hydranal Coulomat AG, Sigma-Aldrich (Barcelona, Spain)) as reagent. The analysis was performed in triplicate and the results averaged.

The acid value (*AV*) was determined according to the ISO 2114:2000 standard. Approximately 2 g of CG were dispersed in 50 mL of ethanol in a 100 mL Erlenmeyer flask. Titrations were conducted using 0.1 N NaOH solution and the end point determined using a digital pH meter (HI 2211 pH/ORP−Hanna Instruments), equipped with a HI 1043B probe. The number of milligrams of KOH required to neutralize the acid of one gram of sample was calculated using Equation (1).

(1) AV=(A−B)×56.1×N/W

where *A* is the volume of NaOH solution required for titration of the sample (mL); *B* is the volume of NaOH solution required for titration of the blank (mL); *N* is the normality of the NaOH solution; and *W* is the weight of the sample (g). 

The hydroxyl number (*OH_number_*) was determined according to the ISO 14900:2001 standard in which the esterification process is catalyzed by imidazole. Titrations were conducted using 0.5 N NaOH solution and the end point determined using a pH meter. The *OH_number_* was corrected taking into account the *AV* and calculated according to Equation (2).

(2) $$OH_{number}=\left(\left(A-B\right)\times 56.1\times N\right)/W+AV$$

where *A* is the volume of NaOH solution required for the titration of the sample (mL); *B* is the volume of NaOH solution required for the titration of the blank (mL); *N* is the normality of the NaOH solution; *W* is the weight of the sample (g); and *AV* is the acidity of the sample (mg_KOH_·g_CG_^−1^).

### 2.3. Production of PUFs

In the production of PUF, CG, surfactant (4 parts per 100 parts of polyol (PHP—*wt/wt*)), catalyst (3 parts per 100 parts of polyol (*wt/wt*)) and blowing agent (6 parts per 100 parts of polyol (*wt/wt*)) were placed in a polypropylene cup. Note that the amount of water present in the CG was subtracted to the amount of blowing agent added. The mixture was homogenized using an IKA Ost Basic mixer with rotating blades, for about 10 s at 700 rpm. Next, the appropriate amount of isocyanate to obtain a *R_NCO/OH_* = 1.10 (ratio between NCO groups of isocyanate and OH groups) was placed in the polypropylene cup and homogenized again. The *R_NCO/OH_* used in the PUFs production was determined using Equation (3).

(3) $$R_{NCO/OH}=\left(m_{iso}\times {\%}_{NCO}/M_{NCO}\right)/\left(m_{polyol}\times \left(OH_{number}+AV\right)/M_{KOH}\;+\left(m_{H2O}+m_{BA}\right)\times Eq_{H2O}\right)$$

where *R_NCO/OH_* is defined as the number of moles of NCO groups of the isocyanate per OH moles of the polyol and water. *m_iso_* is the mass (g) of isocyanate, %NCO is the quantity of NCO groups in the isocyanate (31.1%) and *M_NCO_* is the molecular weight of NCO group (0.042 g·mmol^−1^). *m_polyol_* is the mass (g) of each polyol. *OH_number_* and *AV* are the hydroxyl number and the acid value of the polyol, respectively (mg_KOH_·g^−1^). *M_KOH_* is the molecular weight of KOH (56.1 mg·mmol^−1^). *m_H2O_* is the mass of water present in the polyol. *m_BA_* is the mass of blowing agent (water) added. Finally, *Eq_H2O_* is the equivalent of OH groups present in the water (111 mmol·g^−1^). In the production of PUF filled with EG, EG was dispersed in the appropriated amount of MDI for 30 min using a Sonics Vibra Cell sonicator, while, in the production of PUF filled with PCM, the PCMs were added to the polyol component.

PUFs formulations are listed in Table 1.

### 2.4. Characterization of PUFs

A KD2 Pro (Decagon Devices) was used to measure the thermal conductivity of the PUFs. Samples were analyzed by introducing the thermal conductivity sensor inside the foams. An 8 min gap between analyses was used to ensure the stabilization of the sensor.

SEM analyses were performed in a SU-70 (Hitachi) scanning electron microscope after vacuum-coating with gold to avoid electrostatic charging during examination and at accelerating voltage of 15.0 kV. 

PUFs specimens (10 × 10 × 10 mm^3^) were cut and weighed to determine the density. Densities were determined dividing the weight of the specimens by their respective calculated volume. The values presented correspond to the average density determined for 10 specimens of each foam. 

An Instron 5966 universal mechanical test analyzer was used to measure the compressive stress of the foams, according to the ASTM D 695 standard. Before analysis, PUFs specimens (10 × 10 × 10 mm^3^) were conditioned at 20 °C and 41% relative humidity, in a chamber with humidity control, for 24 h. Samples were then placed between the two parallel plates and compressed at 10 mm/min up to 30% of compression. The Young modulus was calculated by the slope of the tangent of the linear portion (where elastic deformation occurs) of the stress–strain curve. 

TGA of the PUFs was performed using a SETSYS Evolution 1750 thermogravimetric analyzer (Setaram) from room temperature to 800 °C, at a heating rate of 10 °C·min^−1^ and under oxygen flux (200 mL/min).

Differential scanning calorimetric (DSC) analyses were carried out using a Perkin Elmer Diamond DSC from 15 °C to 30 °C at a heating rate of 5 °C·min^−1^ and were used to determine the amount of PCMs in the foams, using Equation (4).

(4) $${\%}_{PCM}=\frac{\Delta H_{foam}}{\Delta H_{PCM}}\times 100$$

where *ΔH_foam_* is the enthalpy of the foams filled with PCMs (J·g^−1^) and *ΔH_PCM_* is the enthalpy of the PCM (J·g^−1^). The DSC analyses were also conducted on the base, middle and top of the foams to evaluate the dispersion of the PCMs along the foams (Table 2), and the average of the three measurements was determined using Equation (5).

(5) $$Entalphy=\frac{\Delta H_{base}+\Delta H_{middle}+\Delta H_{top}}{3}$$

where *ΔH_base_*, *ΔH_middle_* and *ΔH_top_*, are the enthalpies of the base, middle and top of the foams, respectively (J·g^−1^).

### 2.5. Numerical Simulation

The numerical simulations were carried out to evaluate the thermal performance of each foam composition in the form of a single panel, according to their thermal properties. To calibrate and validate the numerical models, the set-up scheme and main results of an experimental testing campaign carried out on two known PUF panels (one with and another without PCM) were used. 

**Figure 1:**
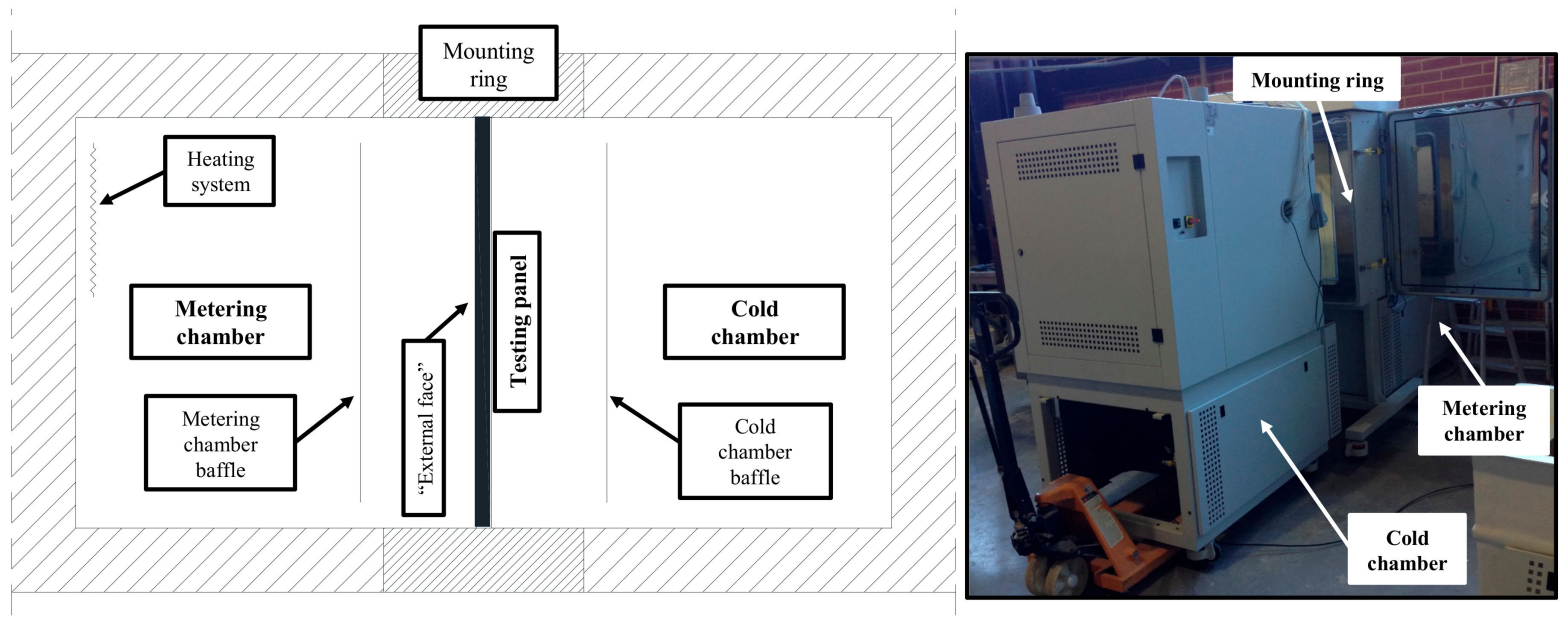
Scheme of the experimental setup adapted from [34].

The modeled PUF panel is considered centered and fixed into a mounting ring that is positioned in between, and divides two climatic chambers (the cold chamber and the warm chamber considered the metering chamber). The modeled setup follows the experimental testing apparatus defining three regions: the cold chamber, the mounting ring where the panel is positioned and the warm chamber, as shown in Figure 1. The chamber walls are composed of: an inner steel sheeting, thick and dense rockwool insulation and an external zinc sheeting and protection. The thickness of these three layers are 1.5 mm, 10 cm and 1.5 mm, respectively.

The materials properties that constitute the climatic chambers were taken from the technical specifications of the chamber manufacturer and the thermal properties of the PUF panels from experimental data (see Table S1), previously determined [34]. All definitions of the numerical models were based on these properties. Other relevant properties of the PCM models are their phase change temperature range. The phase change temperature range of PUF-PCM5.0 is between 23.57 and 26.48 °C and of PUF-EG1.00-PCM5.0 is from 24.29 to 26.80 °C.

**Figure 2:**
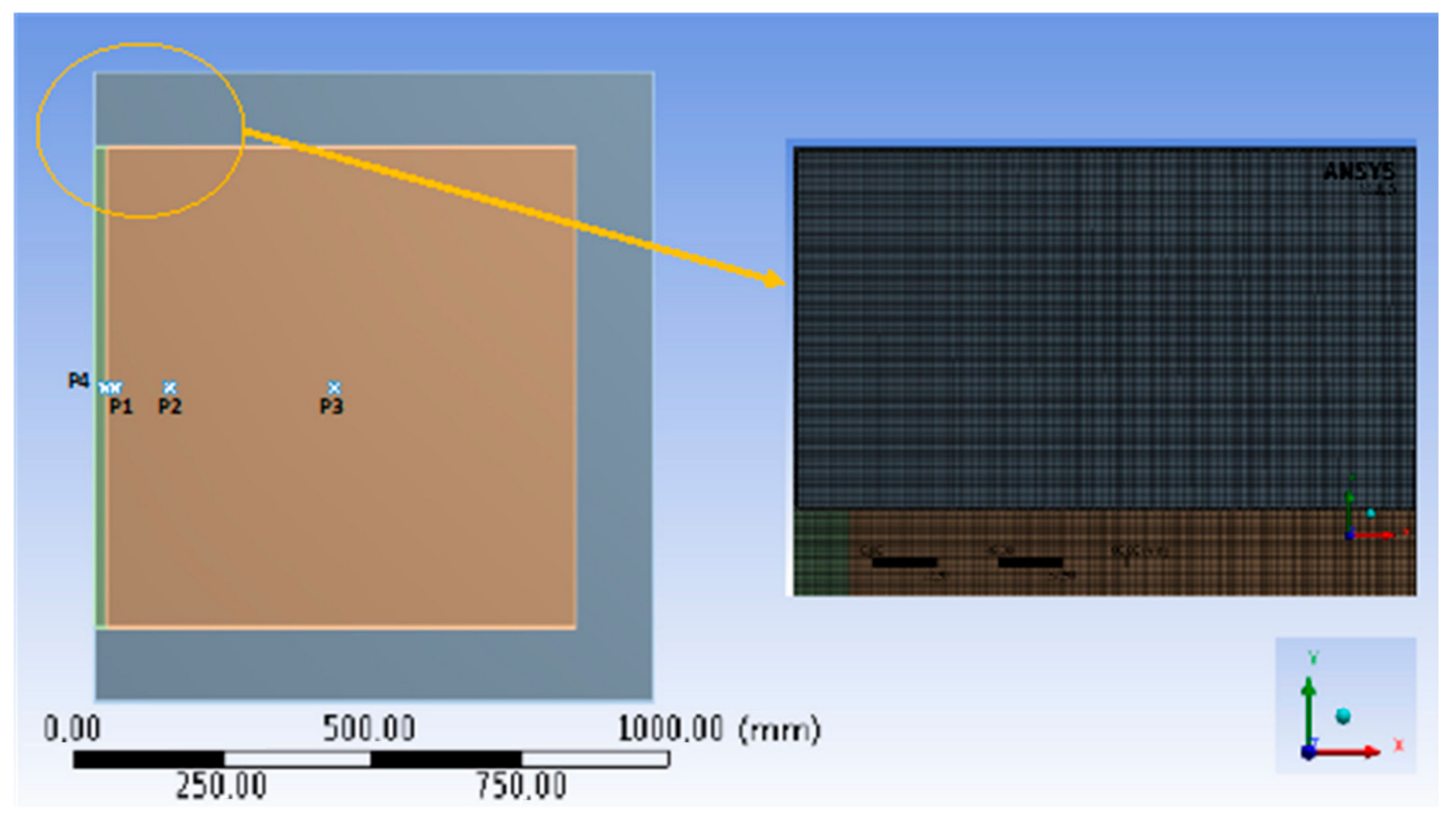
Numerical model (including monitored points/probes).

The 2D geometry of the numerical models was built resourcing to the Solidworks software—as plain surfaces—and the modeled file was exported to ANSYS DesignModeler. To reduce computational time and computer requirements, the cold chamber was not considered and the metering conditions imposed by the warm chamber were applied to the external face of the different PUF panels, as shown in Figure 2.

The temperatures of the metering chamber (warm) were monitored continuously during the imposed cycles, at three points. These temperature probes are located at mid-height of the chamber and considering the position from the external face (warm chamber side). The *x*-axis positions are 20, 249 and 504 mm for points P1, P2 and P3, respectively, as shown in Figure 2. In addition, one more point was added in the middle of the testing panel (point P4) to analyze the PCM liquid fraction. The metering conditions used on the external face (warm side chamber) was the imposed temperature profile (presented in Figure S1), which was considered adequate to assess the PUF panels performance, based on the PCM melting range.

### 2.6. Numerical Validation of the PUF Panels

The numerical calibration was carried out using the results from the two PUF panels tested previously: PUF-REF (without PCMs) and PUF-PCM5-REF (with PCMs) (see Figure S1) [34]. The data from the experimental testing, used as inputs for the numerical models, were the imposed temperature profile (considered on the external face) and the room air temperature variation on the other external boundaries (around the chambers in laboratory conditions with very low temperature fluctuation around 18 ± 2 °C). The imposed temperature is defined as a trapezoidal temperature wave that ranges between 12 °C and 52 °C (blue line in Figure S1), with a period equal to a one day cycle with 6 h steps and a total number of four days. The final output is the temperature profile in the warm chamber that is the average temperature taken from the PT100 probes positioned in the same position that is identified in Figure 2.

The comparison between the numerical models and the experimental testing presented a good agreement with a correlation factor (R^2^) of 93% and 90% for the average values from the PUF-REF and PUF-PCM5-REF panel cases, respectively.

### 2.7. Numerical Testing

Once the model was validated with experimental data, to evaluate the thermal performance of other PUF panel compositions (with different PCM percentages and expandable graphite—designed as PUF-EG1.00-PCM5.0 model), parametric simulations were made. For the new additional simulations, the imposed temperature profile T2 was reconsidered, similar to the previous one, however with a lower thermal amplitude (temperature ranges between 15 and 35 °C). Each time step of the numerical model represents 5 min, so the equivalent of four testing days totals 1152 time steps.

## 3. Results and Discussion

The aim of this study was to enhance the thermal comfort performance of CG derived PUFs, via the incorporation of PCMs aiming at the development of sustainable materials which can contribute to the reduction of the use of non-renewable resources and increase energy savings. First, FTIR analysis was used to monitor the formation of the urethane linkage, as a result of the reaction between the NCO groups of isocyanate and OH groups of CG as well as to monitor the extent of this reaction for the different formulations (see Figure S2). To overcome the low thermal conductivity of the neat foam and improve the efficiency of PCMs, EG was added to the formulation. The amount of EG was optimized by studying its influence on the thermal conductivity of PUFs. In parallel, the effect of PCMs on the morphology, mechanical properties and on the enthalpy of PUF was also assessed. Finally, the selected amounts of EG (1%) and PCM (5%) were used to produce a PUF composite. Considering that the PCM used in the present study was crosslinked, and no leakage of the PCM in liquid state was detected when handling the composite foams or during their characterization, no studies regarding this issue were carried out. 

### 3.1 Thermal Conductivity

**Figure 3:**
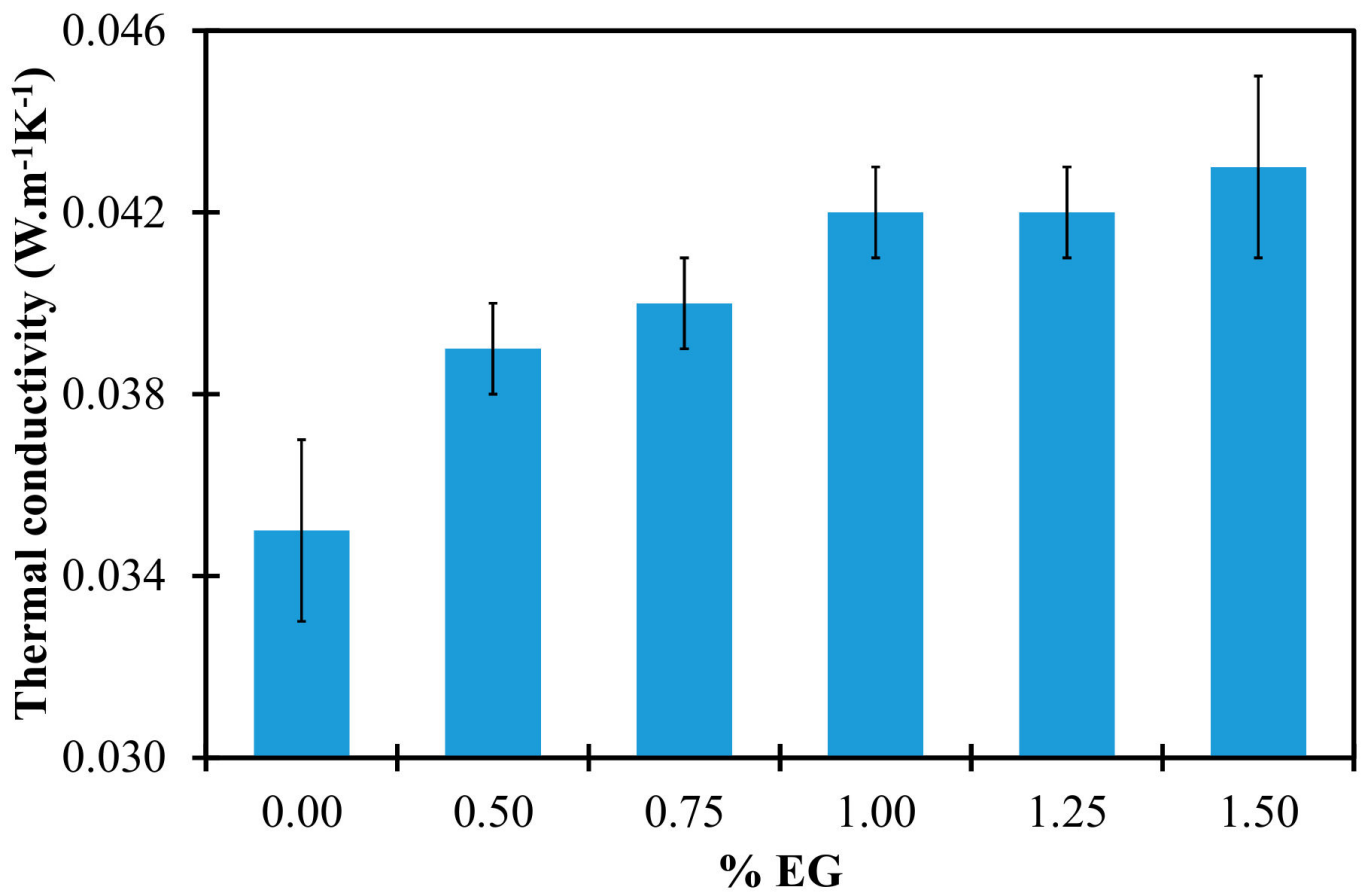
Thermal conductivity of PUF and PUF-EG composites.

PUFs are essentially used in insulation applications. In that sense, the thermal conductivity (*k*) is a property of paramount importance which is related to the foams density, the ratio of open/closed cells and the thermal conductivity of the gas used as blowing agent. However, EG itself has a high thermal conductivity (0.290 W·m^−1^·K^−1^) so its presence was expected to increase the PUFs thermal conductivity. In Table 3 and Figure 3, the neat foam (PUF) has a thermal conductivity of 0.035 W·m^−1^·K^−1^. In turn, the thermal conductivity of PUFs filled with EG increased up to 0.042 W·m^−1^·K^−1^ at 1% (*wt/wt*) of EG. This increment of thermal conductivity resulting from the addition of EG is in agreement with results previously reported in the literature [35]. Above this value, a plateau is reached which could be associated with the formation of an EG network hence, there is no further benefit in adding any more EG. For this reason, 1% (*wt/wt*) of EG was chosen to improve the action of PCMs in the composite foam. 

On the order hand, in Table 3, the addition of PCMs does not have a significant influence on the thermal conductivity of the ensuing foams, being the slight increase of the thermal conductivity related to the increase of the density of the foams.

### 3.2 Morphology

**Figure 4:**
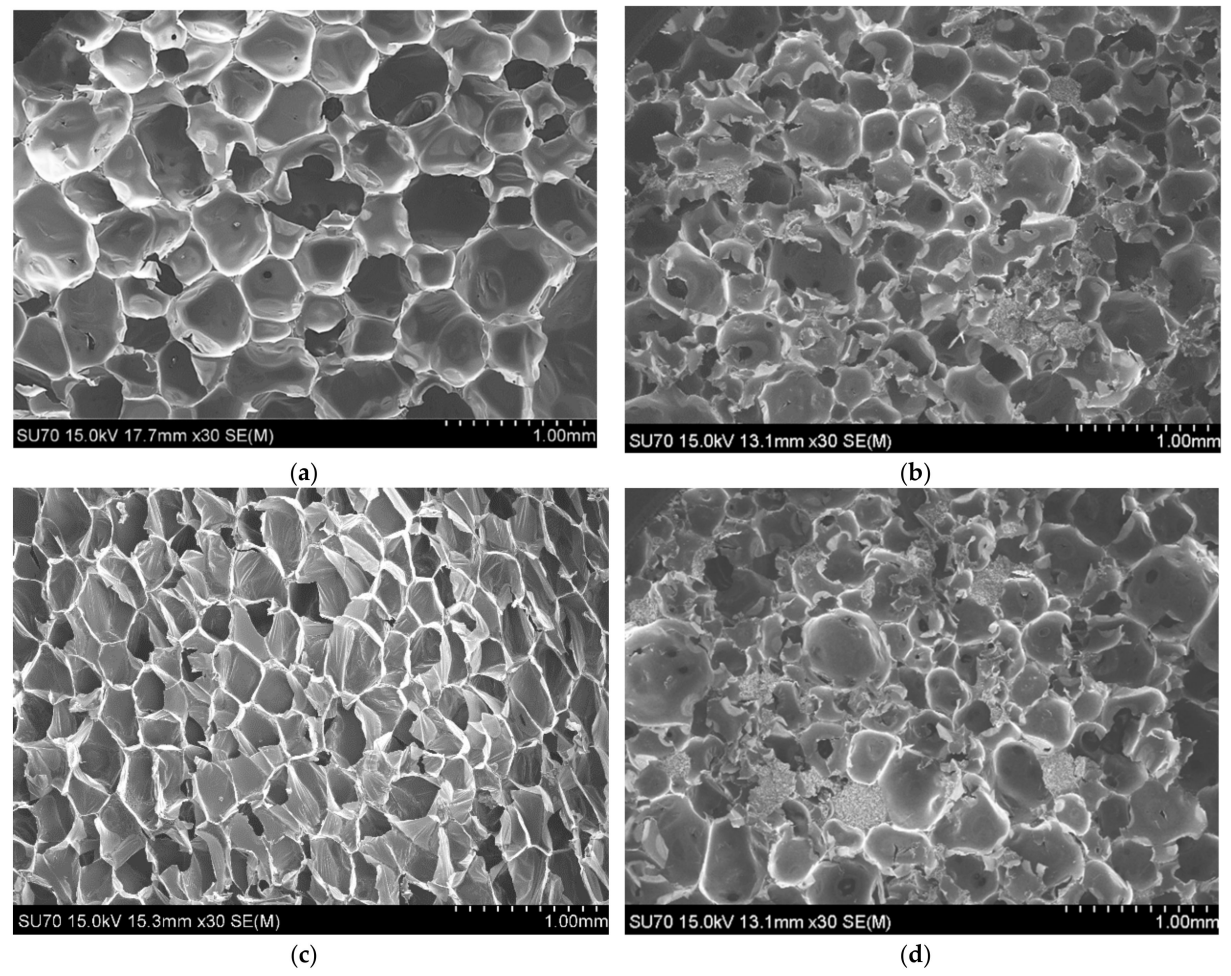
Micrographs of: PUF (**a**); PUF-PCM5.0 (**b**); PUF-EG1.00 (**c**); and PUF-EG1.00-PCM5.0 (**d**).

SEM analysis is an important and versatile tool to inspect the foams’ structures. During mixing, air bubbles are usually introduced in the reaction mixture and act as nucleation sites for the blowing gas generated from the reaction between isocyanate and blowing agent. The bubbles grow resulting in a closely packed network of bubbles responsible for the typical cellular structure of PUFs [36]. The cellular structures of PUFs were observed using a scanning electron microscope at a magnification of 30×, as shown in Figure 4 and Figure S3.

In Figure 4, the neat foam presents the typical cellular structure of PUFs. Moreover, in Figure 4 and Figure S3, the presence of EG does not seem to cause any major disruption of the morphology of the foams. In turn, it is clear that the presence of PCMs increases the heterogeneity of the size and shape of the foam porous structure. The irregularity of the cellular structure of the foams filled with PCMs results from the interactions that PCMs establish with the polymer matrix during bubble nucleation and stabilization stages which affect the foaming process [37]. In fact, similar cases have been reported by others in the literature [38]. For this reason, only 5% (*wt/wt*) of PCMs content was used. Based on the results obtained for PUF-PCM and PUF-EG composites, the foam PUF-EG1.00-PCM5.0 was prepared and its morphology is shown in Figure 4d.

### 3.3. Density

Density is an important property of foams because it affects their thermal and mechanical properties, among others [39]. The density of foams depends on the quantity of gas released during the blowing agent/isocyanate reaction, and on the quantity and nature of the surfactant used [28,40,41]. In addition, the incorporation of a filler (EG, PCMs or both) can affect the foams’ densities since they are denser materials their effect can prevail over the other effects. Moreover, it is quite common to find that the incorporation of fillers can affect foam density due to the interference of the solid particles with the polymer and foaming reactions. The results summarized in Table 3 confirm that the increase of density is related with the increase of the filler content and are in agreement with the results reported in the literature [38,42,43].

### 3.4. Mechanical Properties

**Figure 5:**
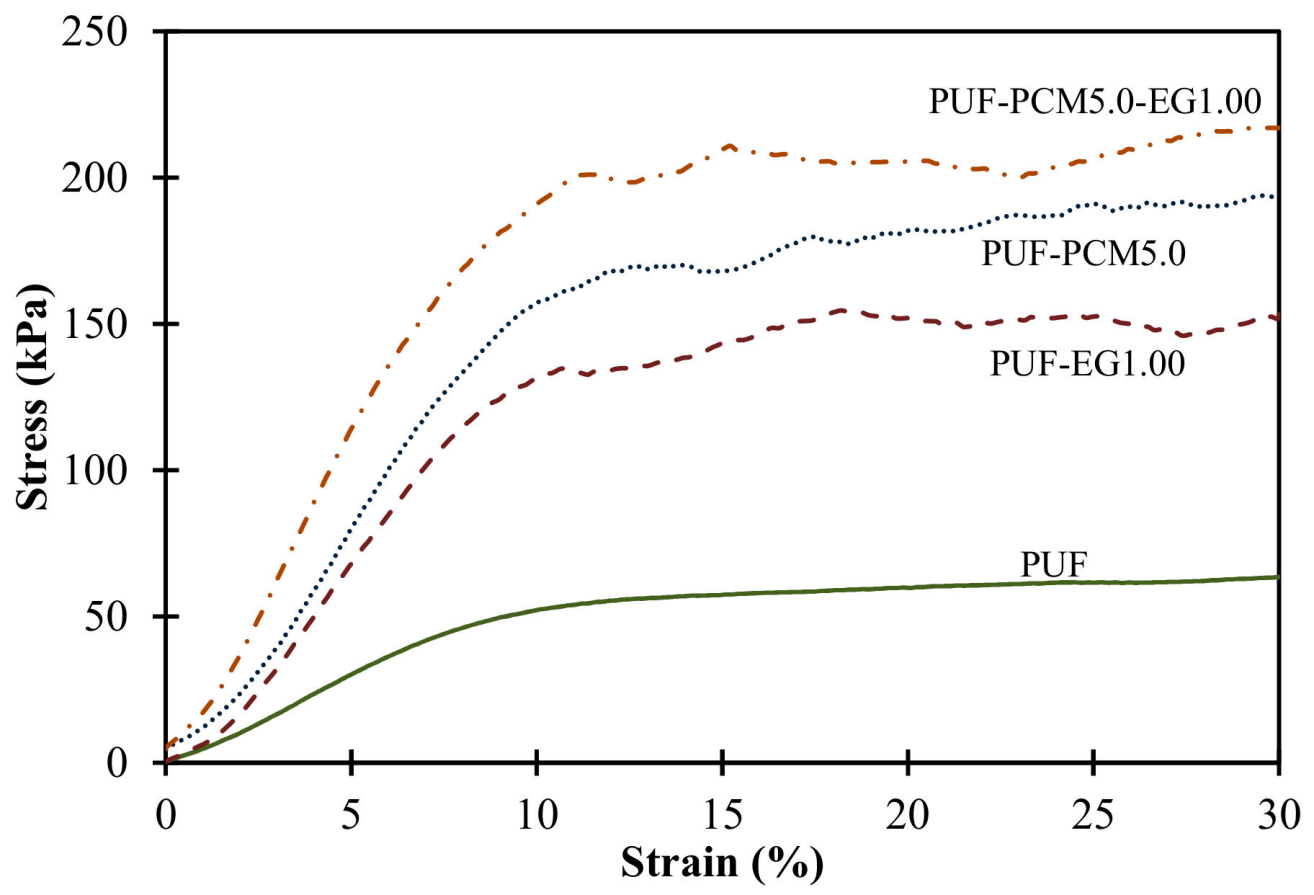
Compressive stress–strain curves of PUF, PUF-EG1.00, PUF-PCM5.0 and PUF-EG1.00-PCM5.0.

Compressive tests were performed to study the mechanical properties of the PUFs and the results obtained are presented in Figure 5, Figure S4 and Table 3.

The mechanical properties of PUFs depend primarily on the formulation and on the cells’ morphology. Thus, changes in the ratio of height to diameter of cells have a major impact. In Figure 5, all compressive stress–strain plots of PUFs show: (*i*) a first linear region which corresponds to the elastic response of the material; and (*ii*) a second region in which the curves present a plateau due to the material deformation, associated with the plastic deformation and/or rupture of the cell walls. Nevertheless, some differences can be observed between the samples. The addition of fillers to PUFs is known to have variable effects depending on the percentage of loading, the size of the particles and whether the fillers are partially incorporated into the cell walls, or between them [42]. Furthermore, the reaction between the isocyanate and the functional groups on the EG surface can lead to a reduction of crosslinking density of the polymer matrix, which affects the mechanical properties of the ensuing composites (see FTIR discussion in supporting information). Moreover, in Table 3, the Young modulus (*E*), toughness and compressive stress (*σ*_10%_) are related with the increase of EG content, as well as with the increase of PCMs content. As the results show, the filler-reinforcing effect increases the slope of the linear region as well as the maximum stress, i.e., it enhances the stiffness of the foams. Besides static mechanical analyses, dynamic mechanical analyses (DMA) were carried out (see Figure S5) and from the results obtained it is concluded that the behavior of PUF-EG1.00 and PUF-EG100-PCM5.0 is more similar than that observed for the stress–strain tests.

### 3.5. Thermogravimetric Analysis

**Figure 6:**
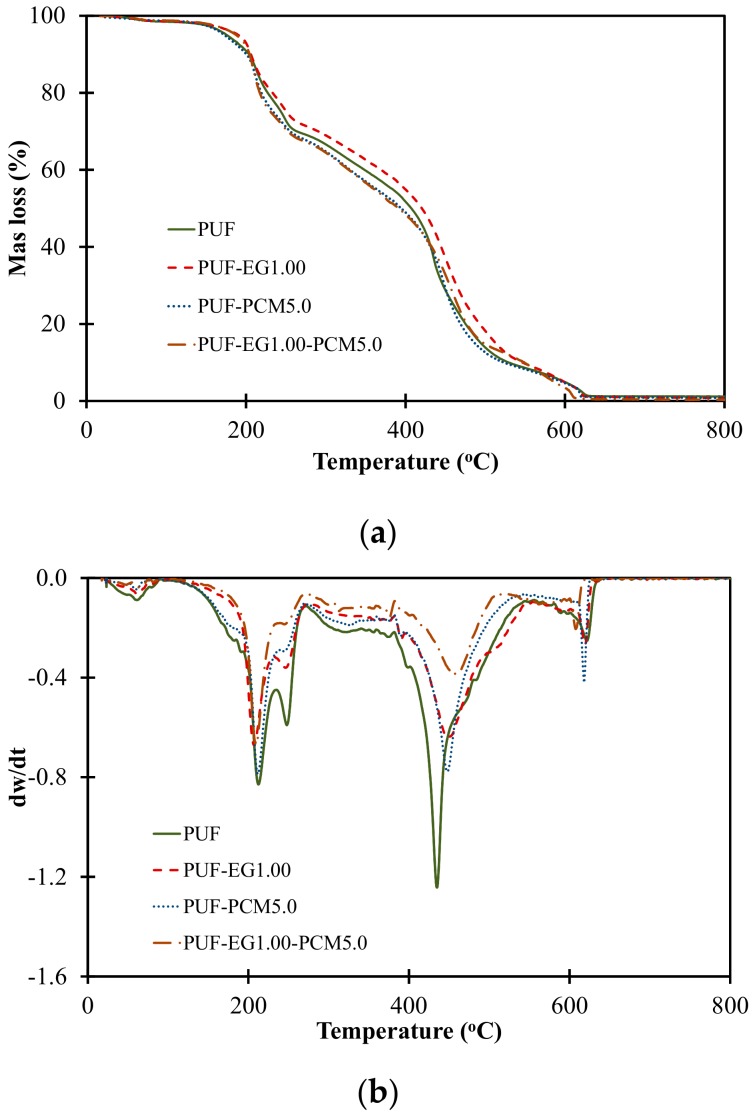
Thermal degradation (**a**); and dw/dt (**b**) of PUF, PUF-EG1.00, PUF-PCM5.0 and PUF-EG1.00-PCM5.0.

Typically, the thermal degradation of PUFs involves the degradation of the hard segments, followed by the degradation of polyol segments, and at lower temperatures the release of some volatile components. The thermal degradation of these samples was investigated by TGA and Figure 6 displays the corresponding TGA curves of PUF, PUF-EG1.00, PUF-PCM5.0 and PUF-EG1.00-PCM5.0. 

In Figure 6, the decomposition of PUFs is characterized by a very small weight loss at around 100 °C due to the release of residual water, followed by two main decompositions steps: one around 210 °C, related to the thermal decomposition of the hard segments (e.g., urethane groups) and a second, around 430 °C, related to the soft segments. The differences detected regarding the rate of decomposition of the soft segments for the PCM foams can be ascribed to the poor dispersion of the fillers and changes of the crosslinking density [44]. This is in agreement with the SEM images where it is clearly visible that the presence of PCMs increases the heterogeneity of foam morphology which is also associated with the effect of PCMs on the polymerization reaction (see Figure S3) and therefore the crosslinking density. The TGA results also suggest that the presence of EG reduced the weight loss of PUFs in the initial stage of degradation. This can be attributed to the barrier effect provided by EG which reduces both the heat and oxygen fluxes toward the polymer surface, which limits the weight loss rate. Finally, Figure 6 reveals small differences in the second decomposition step which can also be attributed to the poor dispersion of the fillers within the cellular structure and differences in crosslinking density, as previously discussed [44]. 

### 3.6. Differential Scanning Calorimetry Analysis

**Figure 7:**
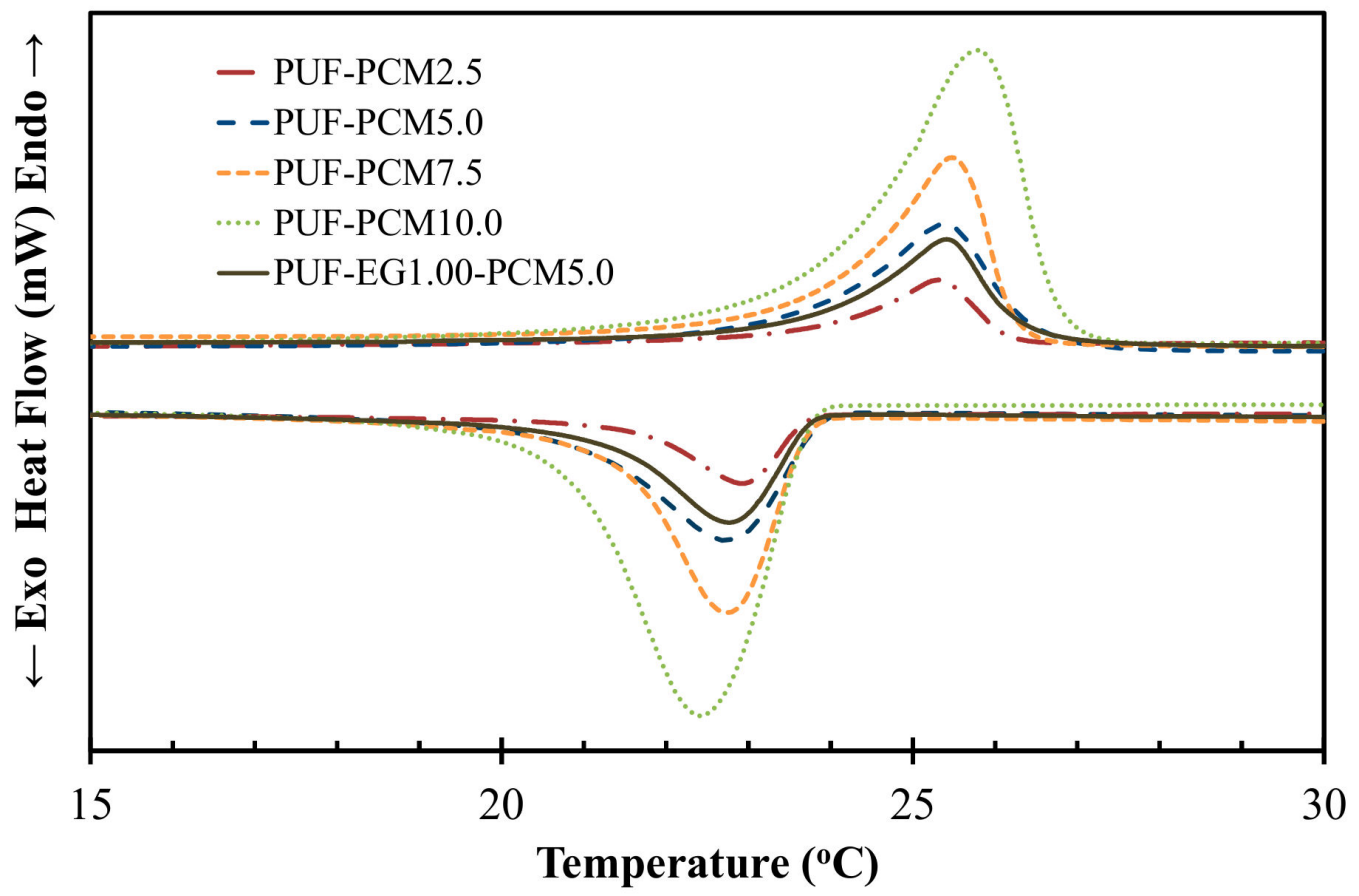
DSC curves of foams.

To study the phase change behavior of the PCMs in the foams, PUFs filled with different PCM contents were analyzed by DSC. The DSC analyses were conducted on the base, middle and top of the foams, to evaluate the dispersion of the PCMs along the direction of the foam expansion and the values presented in Table 2 correspond to their average. DSC curves of PUFs are shown in Figure 7 and the corresponding enthalpy data are summarized in Table 2.

The DSC analyses of the foams revealed well-performed endothermic and exothermic enthalpy changes during heating and cooling between 15 and 30 °C. Table 2 summarizes the observed phase transition intervals as well as the enthalpy values during heating. In each case, the phase transition interval of the foams coincides with the phase transition interval of the PCM. Moreover, considering the low standard deviation of enthalpies presented in Table 2 (which correspond to the average of the values obtained for samples taken at three different points of each foam), it can be concluded that PCMs are reasonably well distributed along the direction of foam’s expansion. Nevertheless, as shown by SEM, within the cellular structure, that is not the case. In Table 2 and Figure 7, it can also be seen that increasing the amount of PCMs, increased the enthalpy of the resulting composites, as expected. Finally, comparing the DSC data of PUF-PCM5.0 and PUF-EG1.00-PCM5.0, it can be seen that the distribution of PCMs along PUF-EG1.00-PCM5.0 is more heterogeneous (higher standard deviation). Furthermore, PUF-PCM5.0 presents similar enthalpy (similar amount of PCMs) but, on the other hand, the initial, maximum and final temperature of the peak of PUF-EG1.00-PCM5.0 are reached at slightly higher temperatures than in the case of PUF-PCM5.0, due to the effect of EG.

### 3.7. Numerical Simulation

**Figure 8:**
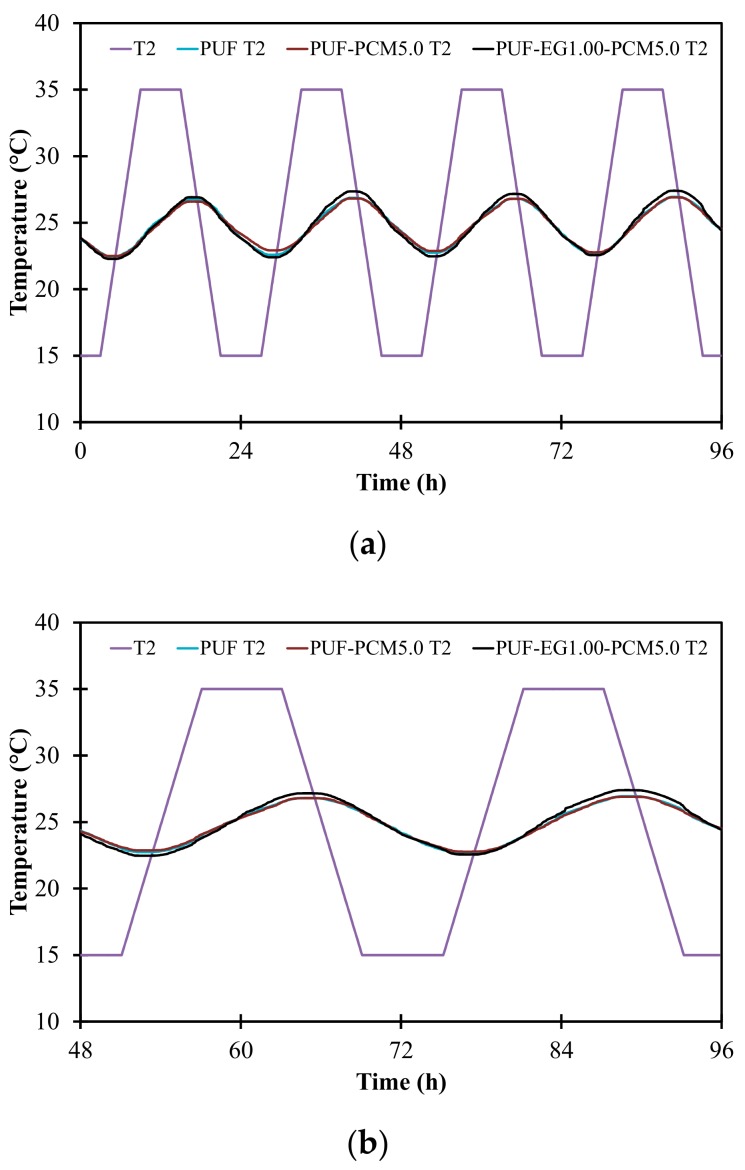
Results of the numerical simulation for: four-day cycles (**a**); and the last two cycles (**b**).

As mentioned in the Introduction, due to the costly action of incorporating PCMs, it is not viable to load PUFs with high amounts of PCMs at large scale. Moreover, their presence can jeopardize the cellular structure of the resulting composite foams and consequently their mechanical properties, as well as further aggravate their reaction to fire. Therefore, numerical simulations were developed to optimize the quantity of PCMs to attenuate the temperature swing in the range of operational thermal comfort conditions (between 20 °C and 25 °C) with the best environmental contribution. The numerical simulations were carried out using a single layer panel composed by each foam composition, using the thermal and physical properties measured. In Figure 8, the temperature results measured in P2 (T2 is the temperature profile at point P2 (see Figure 2)) from each numerical model (PUF, PUF-PCM5.0 and PUF-EG1.00-PCM5.0) are presented. These temperature profiles are the average value of the point/probes monitored inside the warm chamber (see Figure 1).

**Figure 9:**
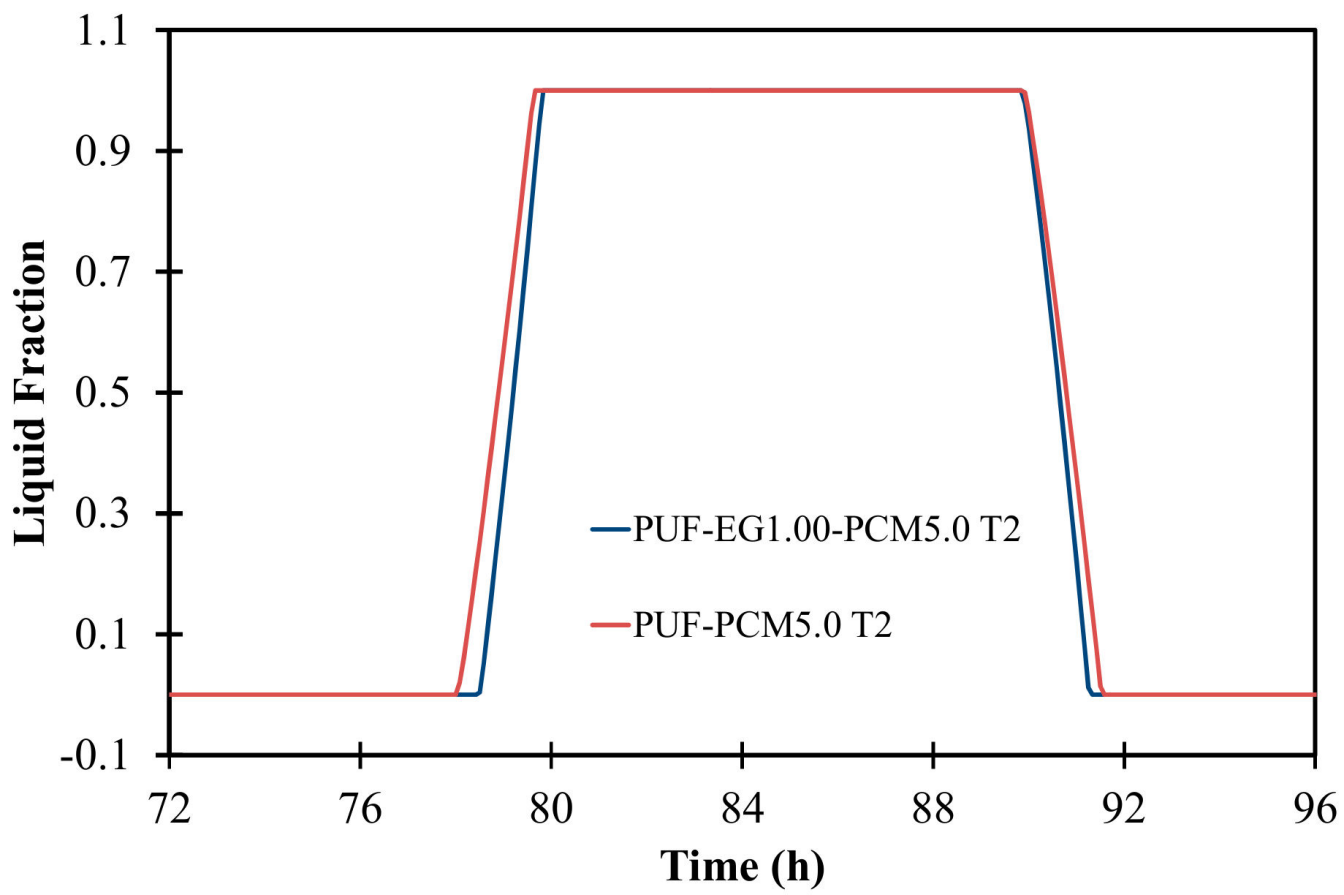
Liquid fraction behavior for the last day cycle.

The temperature profile (T2) of PUF (without PCM) and PUF-PCM5.0 (with PCM) are very similar when comparing the two last cycles. The previous cycles were not considered since they are stabilizing cycles and the numerical modeling results could be misleading, therefore the analysis was based on the last two cycles (see Figure 8b). Considering the imposed temperature T2, and comparing the three testing models (PUF; PUF-PCM5.0 and PUF-EG1.00-PCM5.0), the main conclusions taken from the analysis of Figure 8 are: (*i*) PUF-PCM5.0 and PUF models present similar thermal profiles and thermal amplitudes (4.41 °C vs. 4.32 °C); (*ii*) the PUF-EG1.00-PCM5.0 has a slightly higher thermal amplitude 5.07 °C, as would be expected due to the incorporation of EG; (*iii*) the minimum temperatures are similar for all models, i.e., between 22.27 °C and 22.49 °C (lowest for PUF-EG1.00-PCM5.0 and highest for the PUF-PCM5.0); (*iv*) the maximum temperature is similar for PUF and PUF-PCM5.0 about 26.80 °C, however, for PUF-EG1.00-PCM5.0, it is 0.60 °C higher (27.40 °C); and (*v*) the effect of the incorporation of PCM is not clearly observed from the numerical modeling results. However, further analyses led the authors to look into the PCM liquid fraction to assess the storing and releasing rate of the latent heat energy and in Figure 9 is shown the liquid fraction behavior of the PCM for the last temperature cycle (probe position on *x*-axis 10 mm).

Considering the results presented in Figure 9, there are some additional conclusions that can be stated: (*i*) in both models that incorporate PCM (PCM-5.0 and PUF-EG1.00-PCM5.0), the phase change from solid to liquid is very quick; (*ii*) this phenomenon could be justified by the lower latent heat capacity and/or extreme imposed conditions; (*iii*) the PUF-EG1.00-PCM5.0 model reveals the starting of melt slightly later but is totally melted very close to the same time step of the PUF-PCM5.0 model; (*iv*) both models start to solidify at the same time, but the PUF-PCM5.0 model is totally solidified slightly after the PUF-EG1.00-PCM5.0, as expected, due to the influence on the charging and discharging branches. Moreover, even though the latent heat capacity of PUF-PCM5.0 model is 5242 J·kg^−1^·K^−1^ and its specific heat is higher than that of the PUF model (1620 J·kg^−1^·K^−1^ vs. 833 J·kg^−1^·K^−1^, Table S1) both models presented similar peak temperatures and amplitude. However, comparing the thermal conductivity of these two models, the PUF model has a lower thermal conductivity (0.035 W·m^−1^·K^−1^ vs. 0.037 W·m^−1^·K^−1^), as well as a lower density (43.8 kg·m^−3^ vs. 91.9 kg·m^−3^, Table S1). These differences counter-balanced the thermal behavior of these models, so the final result for the numerical analysis is similar. The numerical results of the PUF-EG1.00-PCM5.0 are the most differing amongst the three models. The thermal properties of this model are similar to the PUF-PCM5.0, except the thermal conductivity, which is higher (0.044 W·m^−1^·K^−1^ vs. 0.037 W·m^−1^·K^−1^, Table S1), and the density, which is also higher (91.9 kg·m^−3^ vs. 115.4 kg·m^−3^, Table S1). These differences result from the incorporation of EG and change the capacity of this model to react faster to the imposed conditions. Therefore, the PCM phase change is faster (during the solidification and melting process) and its effect is reduced. Another limitation of the models incorporating PCM is their quantity. For both models, melting and solidifying occur very quickly which is a constraint to obtain the expectable results due to the low latent heat capacity. Higher quantities of incorporated PCM and subsequently higher latent heat would result in a more evident and longer phase change process and liquid fraction behavior however, as discussed before, the PCM content was limited to 5% due to its impact of the PUFs’ properties. Since the models that consider the incorporation of PCM show higher thermal conductivity, this balances out the expected PCM effect, after the total phase change process occurs, which is a disadvantage since the peak temperatures are lower (discharging) and higher (charging).

## 4. Conclusions

In the present study, composite PUFs were produced from CG which was used directly, without any purification step. The use of unrefined CG glycerol, a byproduct of the biodiesel industry, together with the potential ability to enhance the thermal regulation of buildings, make this type of PUFs ecofriendly as they contribute to the reduction of the use of non-renewable resources and to the increase of energy savings. To improve the heat storage capacity of these materials, the foams were filled with PCMs, and to enhance the action of the PCMs, EG was added. It was concluded that the use of PCMs disrupted the cellular structure of the composite foams, and that the incorporation of PCMs and EG increased the stiffness of the ensuing composite PUFs acting as filler-reinforcing materials. However, the incorporation of these fillers, at least in the formulation used, also caused a substantial increase of the thermal conductivity and density of the ensuing foams which limited their thermal energy storage. Although no leakage of the PCM in liquid state was detected when handling the composite foams, or during their characterization, future studies should assess this aspect as it may compromise, or at least limit, their application.

From the numerical simulations, it was observed that the foams presented similar peak temperatures and amplitude, since the addition of EG and PCMs resulted in an increase of thermal conductivity and density, but similar enthalpy. In other words, the foams filled with PCM had higher thermal conductivity and density, which balanced out the expected PCM effect, i.e., compromised thermal regulation. Nevertheless, the analysis of these exploratory results provided important guidelines for further numerical developments, namely the discussion of the PCM quantity and latent heat capacity, the specific density and thermal conductivity of different PUF compositions, position of the PCM in the case of a multilayer PUF solution, panel thickness and amount of EG to use.

## Figures and Tables

**Table 1:** Foam formulations.

Sample ^a^	CG	Isocyanate (PHP—*wt/wt*)	Catalyst (PHP—*wt/wt*)	Surfactant (PHP—*wt/wt*)	Blowing Agent (PHP—*wt/wt*)	EG (%—*wt/wt*)	PCM (%—*wt/wt*)
PUF	100	209	3	4	4.4	0.0	0.0
PUF-EG0.50	100	209	3	4	4.4	1.4	0.0
PUF-EG0.75	100	209	3	4	4.4	2.0	0.0
PUF-EG1.00	100	209	3	4	4.4	2.7	0.0
PUF-EG1.25	100	209	3	4	4.4	3.4	0.0
PUF-EG1.50	100	209	3	4	4.4	4.1	0.0
PUF-PCM2.5	100	209	3	4	4.4	0.0	6.9
PUF-PCM5.0	100	209	3	4	4.4	0.0	14.2
PUF-PCM7.5	100	209	3	4	4.4	0.0	21.9
PUF-PCM10.0	100	209	3	4	4.4	0.0	29.9
PUF-EG1.00-PCM5.0	100	209	3	4	4.4	2.9	14.3

**^a^** Sample Code (PUF-EGX-PCMY): X, *wt*% of EG; Y, *wt*% of PCM.

**Table 2:** DSC results.

Sample	T_1_ (°C)	T_2_ (°C)	T_3_ (°C)	Enthalpy (J·g^−1^)	% PCM
PCM	23.87	26.16	27.51	103.3891	100
PUF-PCM2.5	23.95	25.31	26.16	2.5989 ± 0.1415	2.5 ± 0.1
PUF-PCM5.0	23.57	25.34	26.48	5.2420 ± 0.1266	5.1 ± 0.1
PUF-PCM7.5	23.77	25.41	26.13	7.6177 ± 0.2090	7.4 ± 0.2
PUF-PCM10.0	24.11	25.78	26.74	10.3811 ± 0.3448	10.0 ± 0.3
PUF-EG1.00-PCM5.0	24.29	25.91	26.80	5.1499 ± 0.4821	5.0 ± 0.5

T_1_, initial temperature of the peak; T_2_, maximum temperature of the peak; T_3_, final temperature of the peak.

**Table 3:** Properties of ensuing foams.

Sample	Density (kg·m^−3^)	Thermal Conductivity (W·m^−1^·K^−1^)	Young Modulus (kPa)	Toughness (J·m^−3^)	Compressive Stress σ_10%_ (kPa)
PUF	43.8 ± 1.4	0.035 ± 0.002	643 ± 27	15560 ± 1371	55 ± 2
PUF-EG0.50	74.0 ± 3.7	0.039 ± 0.001	1334 ± 43	29313 ± 1344	95 ± 4
PUF-EG0.75	84.2 ± 2.6	0.040 ± 0.001	1541 ± 59	34500 ± 2027	114 ± 7
PUF-EG1.00	92.9 ± 3.9	0.042 ± 0.001	1739 ± 74	40640 ± 1754	144 ± 8
PUF-EG1.25	84.8 ± 4.6	0.042 ± 0.001	1812 ± 66	42775 ± 2814	152 ± 8
PUF-EG1.50	99.3 ± 5.0	0.043 ± 0.002	1875 ± 89	45075 ± 4738	162 ± 8
PUF-PCM2.5	81.8 ± 2.9	0.036 ± 0.001	1729 ± 57	40280 ± 2132	144 ± 8
PUF-PCM5.0	91.9 ± 4.0	0.037 ± 0.002	2079 ± 91	47467 ± 2605	167 ± 11
PUF-PCM7.5	103.0 ± 6.0	0.038 ± 0.003	2312 ± 85	51960 ± 7412	175 ± 12
PUF-PCM10.0	118.2 ± 7.9	0.039 ± 0.001	2771 ± 117	59983 ± 3068	193 ± 12
PUF-EG1.00-PCM5.0	115.4 ± 5.8	0.044 ± 0.002	2765 ± 113	58856 ± 2163	193 ± 11

## Supplementary Materials

More details regarding the numerical modeling, numerical validation and further characterization data (FTIR, SEM, mechanical tests and DMA) of the composites can be found in the supporting information.
