# Supplementary material for: Thermal Energy Storage and Mechanical Performance of Crude Glycerol Polyurethane Composite Foams Containing Phase Change Materials and Expandable Graphite
Source: Materials (Basel). 2018 Oct 4;11(10):1896. doi: 10.3390/ma11101896 (PMC6212961; doi:10.3390/ma11101896)
Supplement: Supplementary file 1 [file materials-11-01896-s001.pdf]

materials

Supplementary

Thermal Energy Storage and Mechanical Performance of Crude Glycerol Polyurethane Composite Foams Containing Phase Change Materials and Expandable Graphite

Nuno Vasco Gama [1,2], Cláudia Amaral [1,2], Tiago Silva [1], Romeu Vicente [3,4], João Araújo Pereira Coutinho [1,2], Ana Barros-Timmons [1,2,]* and Artur Ferreira [1,5]

1 CICECO—Aveiro Institute of Materials, 3810-193 Aveiro, Portugal; nuno.gama@ua.pt (N.V.G.); claudiaamaral@ua.pt (C.A.); tiagomsilva@ua.pt (T.S.); jcoutinho@ua.pt (J.A.P.C.)
2 Dept. of Chemistry of University of Aveiro, 3810-193 Aveiro, Portugal;
3 RISCO, 3810-193 Aveiro, Portugal; romvic@ua.pt
4 Civil Engineering of University of Aveiro, 3810-193 Aveiro, Portugal;
5 Escola Superior de Tecnologia e Gestão de Águeda, 3750-127 Águeda, Portugal; artur.ferreira@ua.pt
* Correspondence: anabarros@ua.pt

- Numerical modelling

Table S1 - Thermal properties of the chamber walls and PUF panels.

Material	Thickness (mm)	Density ($kg.m^{-3}$)	Thermal conductivity ($W.m^{-1}.K^{-1}$)	Specific heat ($J.kg^{-1}.K^{-1}$)	Latent heat ($J.kg^{-1}.K^{-1}$)
Inner Steel sheeting	1.5	7900	14.9	477	n.a
Rockwool insulation	100	105	0.042	840	n.a
Outer Zinc sheeting	1.5	7140	116	389	n.a
PUF REF	20	43.8	0.035	833	n.a
PUF-PCM REF	20	110	0.038	2000	6160
PUF-PCM5.0	20	91.9	0.037	1620	5242
PUF-EG1.00-PCM5.0	20	115.4	0.044	1666	5150
air inside chambers	-	7833	54	465	-

n.a – Not available

- Numerical validation of the PUF panels models

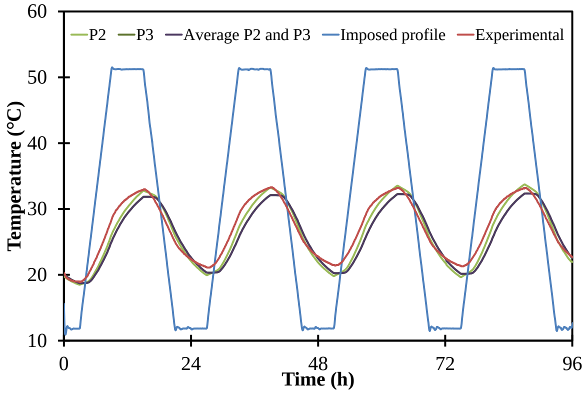

(a)

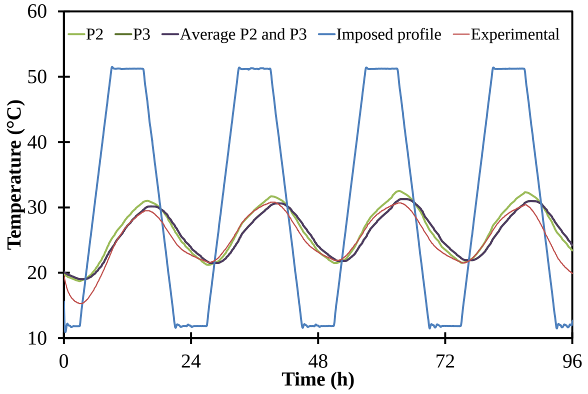

(b)

Figure S1. Temperature curves comparison (experimental and numerical data) of PUF without PCMs (PUF REF) (a) and PUF with PCMs (PUF-PCM REF) (b) used as reference for the numerical modelling

- Monitorization of reaction

FTIR analysis was used to monitor the formation of the urethane linkage, as a result of the reaction between the NCO groups of isocyanate and OH groups of CG as well as to monitor the extent of this reaction. The FTIR spectra of PUFs were collected on a Perkin Elmer FTIR System Spectrum BX Spectrometer equipped with a single horizontal Golden Gate ATR cell. All data were recorded at room temperature, in the range 4000 to 600 cm^{-1} by accumulating 32 scans with a resolution of 4 cm^{-1}. Figure S2 shows the normalized FTIR spectra of PUF, PUF-EG1.00, PUF-PCM5.0 and PUF-EG1.00-PCM5.0.

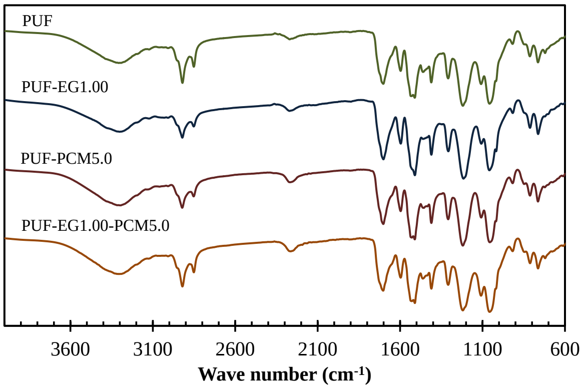

Figure S2. Normalized FTIR spectra of PUFs

Comparing the normalized FTIR spectra of PUFs it can be seen that all present a very similar profile. The band in the 3200–3450 cm^{-1} region is attributed to the non-bonded stretching vibrations of O-H groups and symmetric and asymmetric stretching vibrations of the N-H of the urethane and of urea groups (resulting from the reaction between water and some of the isocyanate groups). The bands between 2950 and 2850 cm^{-1} correspond to the asymmetric and symmetric C-H stretching vibrations respectively. The peak at 1710 cm^{-1} is due to the stretching vibrations of the C=O whilst the nearly overlapped bands between 1540 and 1517 cm^{-1} are attributed to the stretching and bending vibrations of the C-N and N-H of the urethane moieties, respectively. The bands at 1220 cm^{-1} and 1100 cm^{-1} are associated with the C-O stretching vibrations.

The small peak around 2270 cm^{-1}, typical of residual NCO groups, is due to the excess of isocyanate used in the production of PUFs. Also, notice should be made that the presence of fillers (EG, PCM and both) increased the 2270 cm^{-1} peak in the spectra of the corresponding composites. This may be attributed to at least two possibilities: *(i)* the hydroxyl groups on the surface of EG can alter the isocyanate index ($R_{NCO/OH}$) defined as the number of moles of NCO groups of the isocyanate per OH mole of the polyol thus affecting the consumption of NCO groups and *(ii)* the interference of the filler on the rate of the polymerization namely associated with changes in the rheological behavior of the reaction mixture.[1] Both possibilities may lead to a lower crosslinking density and affect the morphology of the foams as well as the thermal and mechanical properties of the ensuing composites. In fact, this can justify the differences on the structure and on the mechanical properties of the resulting foams.

- Morphology of composites

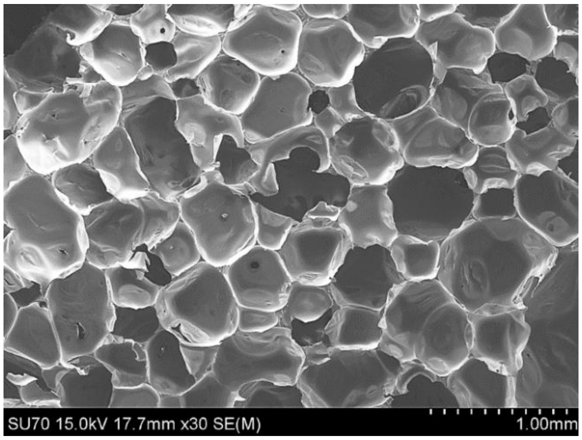
SU70 15.0kV 17.7mm x30 SE(M)
1.00mm

(a)

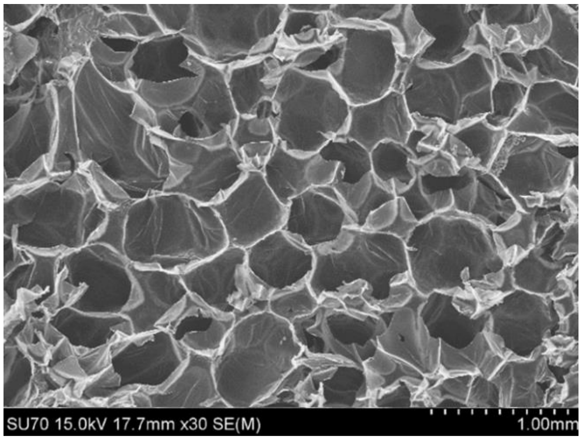
SU70 15.0kV 17.7mm x30 SE(M)
1.00mm

(b)

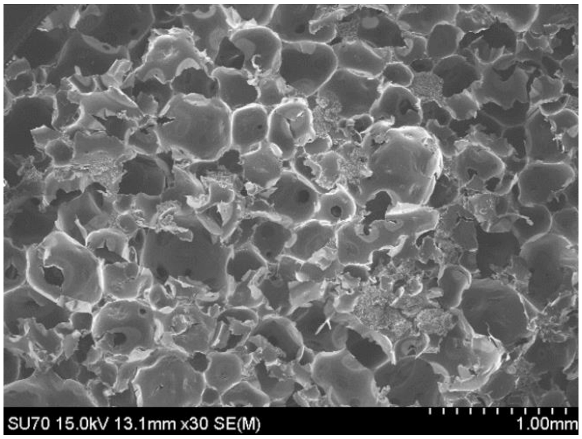
SU70 15.0kV 13.1mm x30 SE(M)
1.00mm

(c)

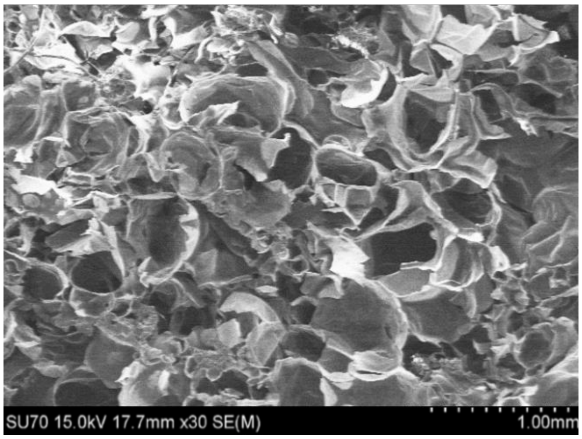
SU70 15.0kV 17.7mm x30 SE(M)
1.00mm

(d)

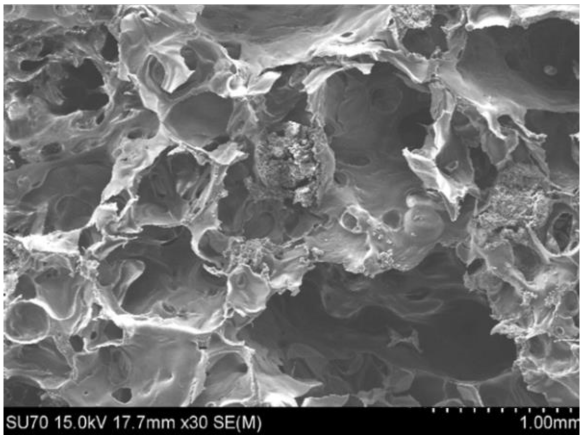
SU70 15.0kV 17.7mm x30 SE(M)
1.00mm

(e)

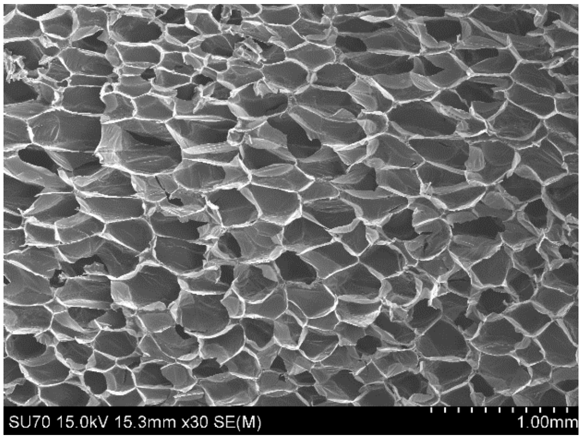
SU70 15.0kV 15.3mm x30 SE(M)
1.00mm

(f)

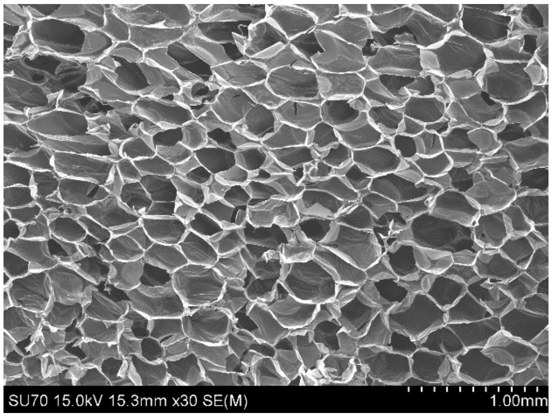

(g)

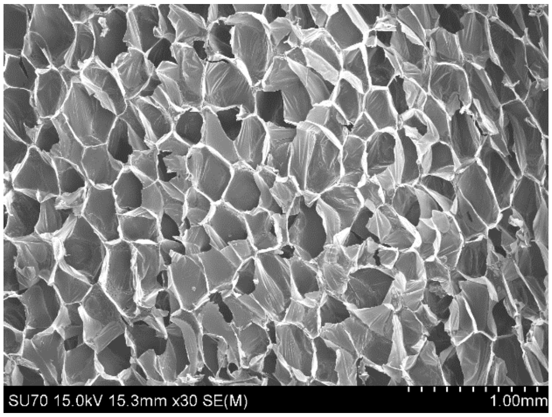

(h)

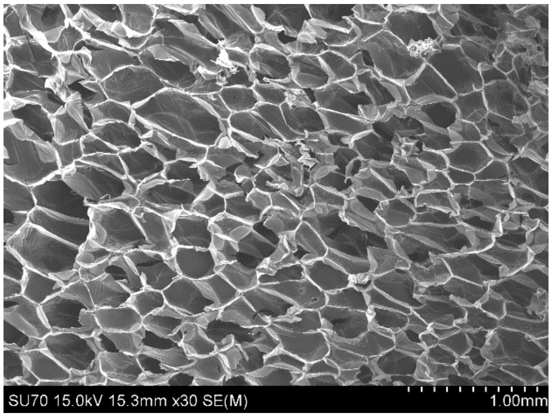

(i)

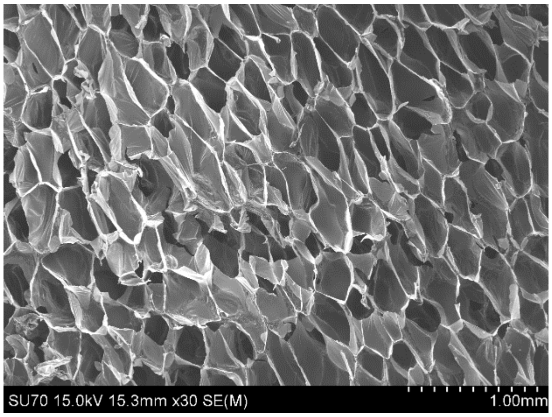

(j)

Figure S3. Micrograph of PUF (a), PUF-PCM2.5 (b), PUF-PCM5.0 (c), PUF-PCM7.5 (d PUF-PCM10.0 (e), PUF-EG0.50 (f), PUF-EG0.75 (g), PUF-EG1.00 (h), PUF-EG1.25 (i) and PUF-EG1.50 (j)

- Mechanical properties of composites

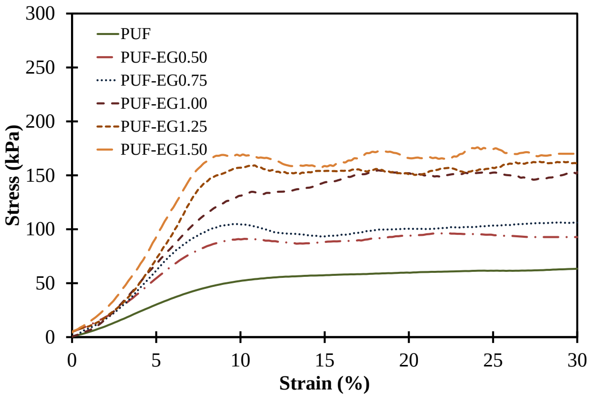

(a)

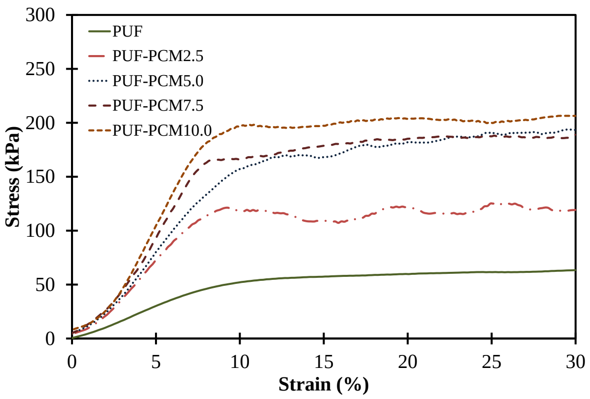

(b)

Figure S4. Compressive stress-strain curves of EG composites (a) and PCMs composites (b)

- Dynamic mechanical analysis

Dynamic mechanical analyses (DMA) were carried out using a Tritec 2000 equipment (Triton Technologies). Samples with dimensions of 10 x 9 x 6 mm^3 were compressed from -55 ºC up to 150 ºC at a constant heating rate of 2 $ºC.min^{-1}$ and at a frequency of 1 Hz.

DMA experiments were carried out in order to obtain further information on the viscoelastic properties of PUFs. Materials such as PUFs typically exhibit two distinct types of behavior depending on the temperature. At low temperatures they exhibit the properties of a glass (high modulus) and at higher temperatures those of a rubber (low modulus). Figure S4 displays the temperature dependence of the storage modulus and tan (δ) of PUF, PUF-EG1.00, PUF-PCM5.0 and PUF-EG1.00-PCM5.0.

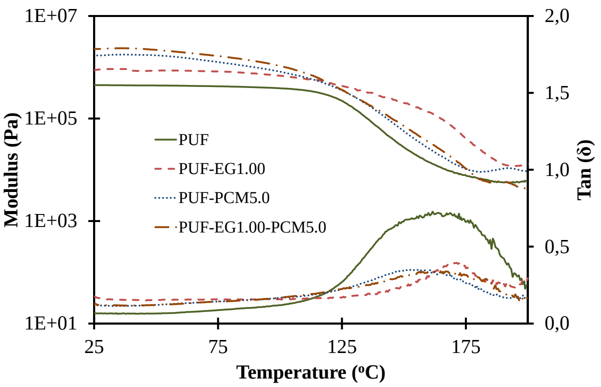

Figure S5. Temperature dependence of the storage modulus and tan (δ) of PUF, PUF-EG1.00, PUF-PCM5.0 and PUF-EG1.00-PCM5.0

Below the glass transition temperature, PUF-EG1.00 and PUF-EG100-PCM5.0 have similar storage modulus around 1.9x10^6 Pa. Interestingly, in this type of dynamic mode of analyses, the behavior of PUF-EG1.00 and PUF-EG100-PCM5.0 is much more similar then that observed for the stress-strain tests.

References

1. Gama, N.; Costa, L. C.; Amaral, V.; Ferreira, A.; Barros-Timmons, A. *Compos. Sci. Technol.* **2017**, *138*, 24–31.

© 2018 by the authors. Licensee MDPI, Basel, Switzerland. This article is an open access article distributed under the terms and conditions of the Creative Commons Attribution (CC BY) license (http://creativecommons.org/licenses/by/4.0/).
